# Multi‐Modal Approach to Salvage: Extracorporeal Shock Wave Lithotripsy and Flexible Ureteroscopy for Encrusted DJ Stent Removal in Renal Allograft—A Case Report and Literature Review

**DOI:** 10.1002/ccr3.70895

**Published:** 2025-09-16

**Authors:** Rao Nouman Ali, Adeel Anwaar, Wajiha Irfan, Farooq Hameed, Inam Ul Haq, Riyan Imtiaz Karamat, Aymar Akilimali

**Affiliations:** ^1^ District Head Quarter Hospital Khanewal Pakistan; ^2^ Punjab Rangers Teaching Hospital Lahore Pakistan; ^3^ Urology Suite Midcity Hospital Lahore Pakistan; ^4^ Combined Military Hospital Multan Pakistan; ^5^ Hijaz Hospital Gulberg Lahore Pakistan; ^6^ Rashid Latif Medical and Dental College Lahore Pakistan; ^7^ Rahbar Medical and Dental College Lahore Pakistan; ^8^ Department of Research Medical Research Circle (MedReC) Goma Democratic Republic of Congo

**Keywords:** allograft, case report, encrusted DJ stent, flexible ureteroscopy, holmium laser

## Abstract

DJ stents are critical for maintaining renal drainage in post‐surgical and obstructive conditions, but they must be removed within 4–6 weeks to prevent serious complications. This case highlights the successful removal of an encrusted, forgotten DJ stent through extracorporeal shock wave lithotripsy (ESWL) in a renal transplant patient, resulting in an uneventful recovery.

## Graphical abstract

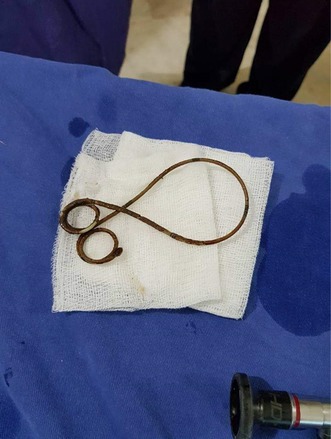

## 1 Introduction

DJ stents have been widely utilized since their invention by Zimskind in 1960 [1]. The morbidity associated with these stents is directly proportional to the duration they remain in the renal tracts; therefore, it is crucial to respect their half‐life and ensure timely removal. If left unnoticed, DJ stents can become encrusted with stones, making removal through conventional cystoscopy and endo‐graspers challenging [2]. In recent decades, there has been a significant increase in DJ stent‐related morbidity, prompting the development of various modalities for removing encrusted stents. Techniques such as extracorporeal shock wave lithotripsy, transureteral lithotripsy, percutaneous nephrolithotomy, and cystolithotripsy are now commonly employed. Depending on the situation, one or more of these methods may be utilized to remove forgotten, encrusted DJ stents [3]. The use of DJ stents in kidney transplant recipients is vital to ensure the patency of the uretero‐vesical anastomosis, with typical removal occurring between 2 and 6 weeks post‐transplant. However, despite close monitoring, various factors such as communication barriers, poor understanding of the condition, financial constraints, prolonged asymptomatic periods, and regional issues can contribute to the challenging scenario of a forgotten, encrusted DJ stent in renal allograft recipients [4]. The removal of such a stent in these patients requires a meticulous approach due to the critical importance of preserving the solitary functioning kidney, which is essential for avoiding the burdens of cumbersome hemodialysis [5].

In this case, our kidney transplant patient presented very late with an encrusted DJ stent. Attempts to remove the stent using conventional methods were unsuccessful due to the entrapment of the upper curl of the DJ stent. Consequently, we employed extracorporeal shock wave lithotripsy and flexible ureteroscopy with holmium laser to successfully remove the long‐retained DJ stent.

## 2 Case Presentation

### 2.1 Case History/Examination

A 32‐year‐old woman from Afghanistan underwent a living‐related renal transplant in 2020 due to end‐stage renal disease secondary to polycystic kidney disease. During the procedure, the Lich‐Gregoir technique was utilized for ureteroneocystostomy, and a 6 French DJ stent was placed to ensure the patency of the uretero‐vesical anastomosis. In December 2023, the patient presented to our urology ward with complaints of dysuria, hematuria, fever, and intermittent vomiting lasting one month. Upon examination, she exhibited tachypnea, tachycardia, and suprapubic tenderness, while the remainder of the physical examination was unremarkable.

### 2.2 Imaging Investigation Findings

**FIGURE 1:**
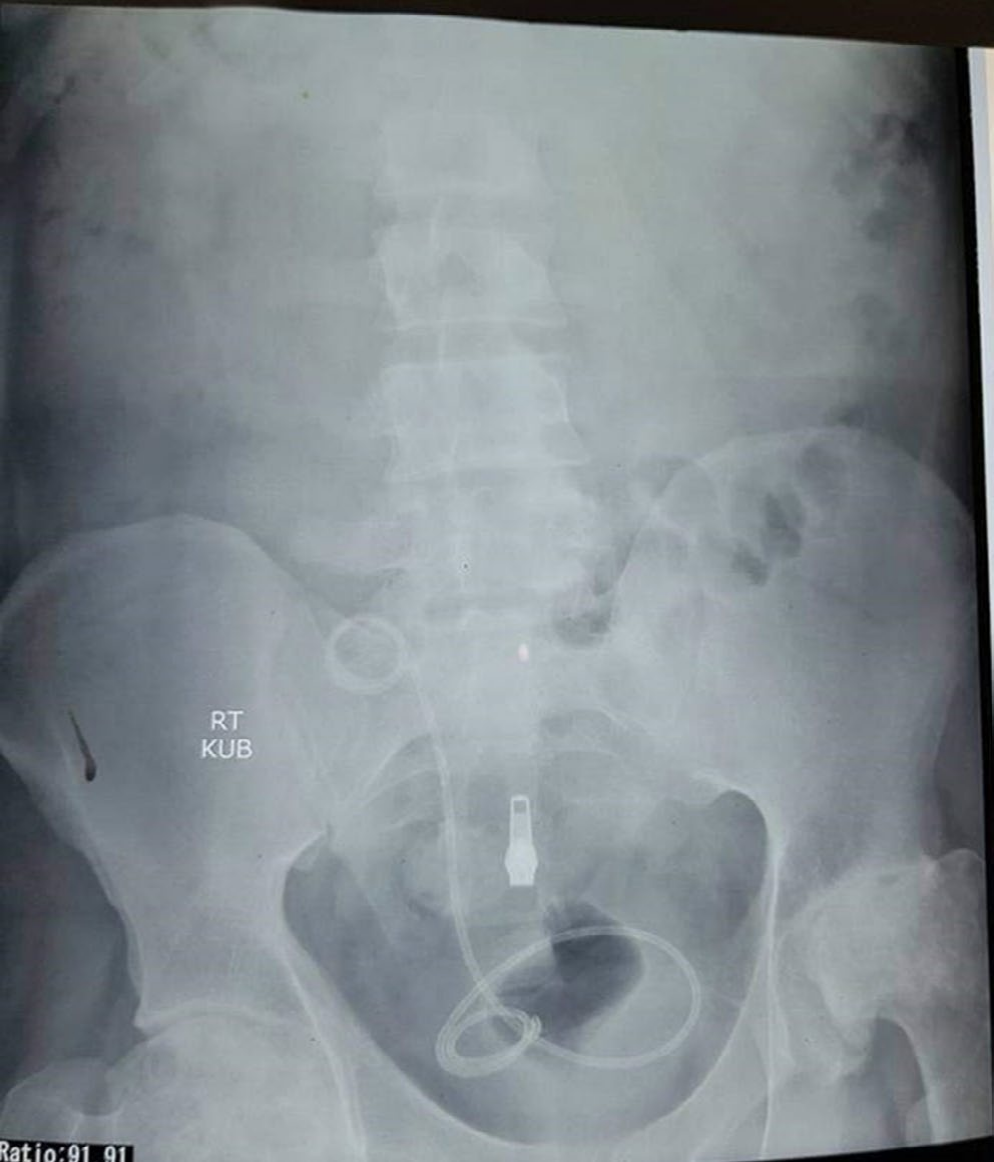
X‐ray KUB.

Routine laboratory investigations revealed the following: hemoglobin of 10 g/dL, white blood cell count of 9000/μL, platelet count of 180,000/μL, serum creatinine of 1.4 mg/dL, and urea of 25 mg/dL, with all other lab results within normal limits. Urinalysis demonstrated the presence of red blood cells (15–20/HPF), white blood cells (10–13/HPF), and trace amounts of calcium phosphate crystals. An ultrasound examination revealed a hyperechoic curl of the DJ stent within the allograft kidney. Same findings were confirmed on X‐ray KUB (Figure 1).

### 2.3 Surgical Management

**FIGURE 2:**
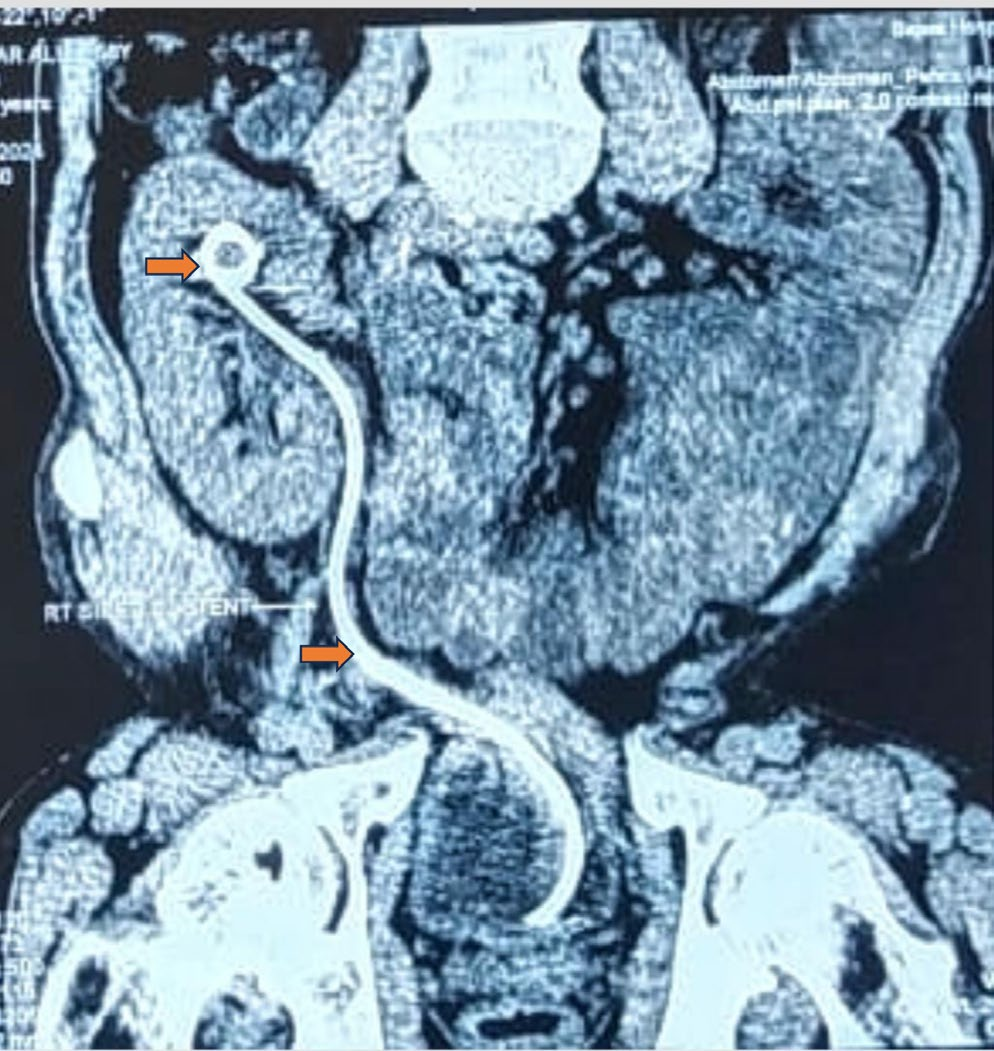
CT scan revealed encrustation at the upper end of the DJ stent.

**FIGURE 3:**
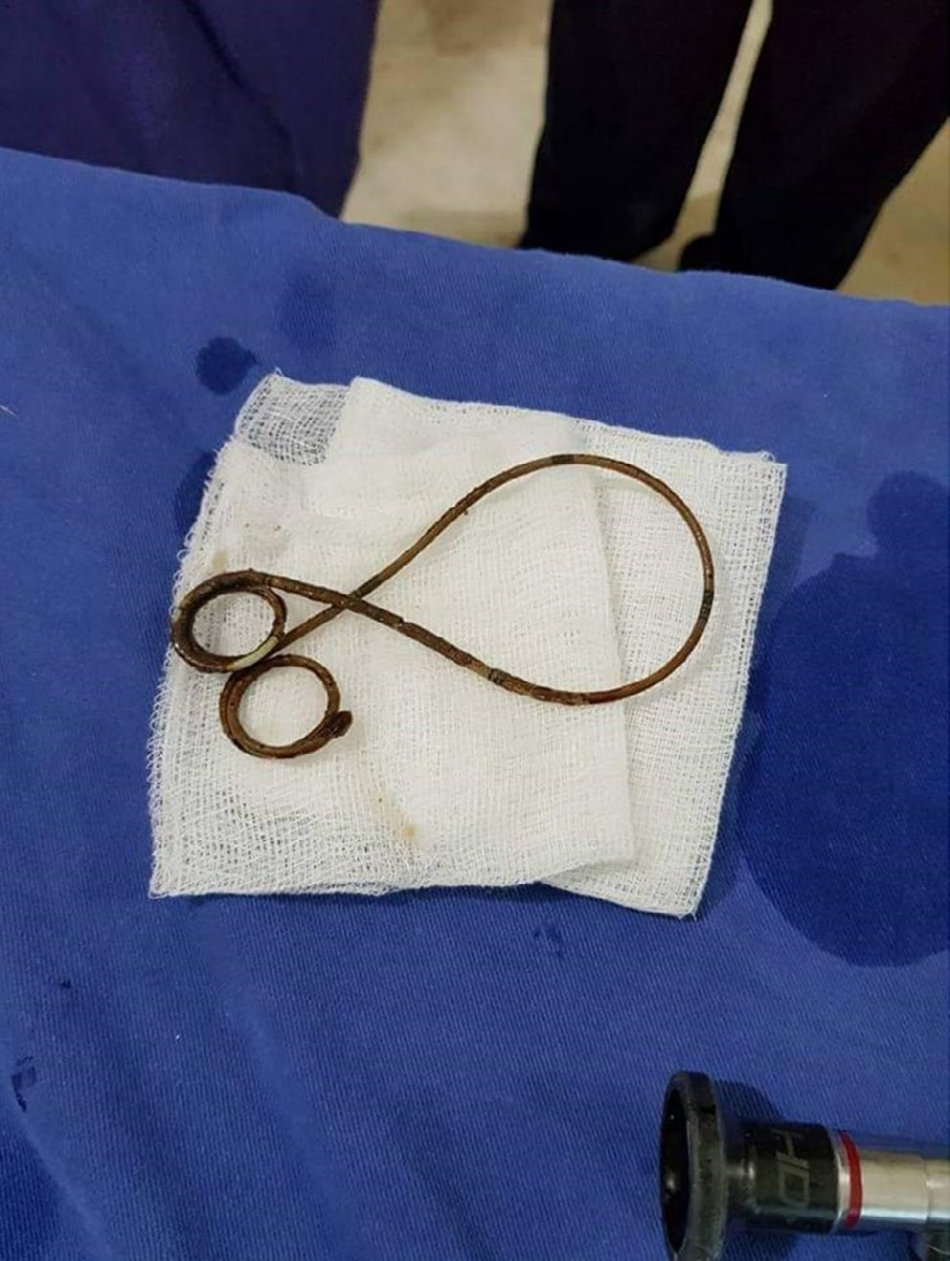
Successfully retrieved the completely encrusted, forgotten DJ stent from the allograft.

The patient was admitted to the hospital, started on antibiotics, and prepared for the removal of the trapped DJ stent via cystoscopy and endo‐grasper. However, we were unsuccessful in retrieving the stent, even though the lower curl of the stent showed minimal encrustation. Subsequently, the patient was transferred back to the ward, where a computed tomography (CT) scan was performed (Figure 2). The CT scan revealed encrustation at the upper end of the DJ stent with 926 Houns Field Units (HU), prompting us to utilize extracorporeal shock wave lithotripsy (ESWL) to address the encrusted upper end of the stent while the patient was under broad‐spectrum antibiotic coverage. The miniPCNL was not considered because of less stone burden, the invasive nature of the modality, and to prevent precious allograft from surgical trauma; hence, it was unanimously decided to utilize extracorporeal shock wave lithotripsy with the help of a latest lithotripter named “Modularis vario” which is an electromagnetic source of shock waves production along with incorporated fluoroscopic guidance. The patient was treated in a supine position, and fluoroscopy was used to locate the upper end double J stent encrustations. There were about 2500–3000 shocks were given per session with a gradual raise in energy levels from 0.1 to 0.9 and increased up to 3.3 for encrustations on the upper and middle portions of the double J stent with a frequency of 60–90 shocks/min utilized in both sessions. After two sessions of ESWL, we again prepared the patient for flexible ureteroscopy, and holmium laser was utilized with the help of 200–272 micrometer laser fiber with low energy settings of 0.6–1 joules to gradually fragment and dust the lower end encrustations of the double J stent; hence, we successfully retrieved the completely encrusted, forgotten DJ stent from the allograft. (Figure 3).

### 2.4 Ongoing Follow‐Up Plan

Typically, renal transplant recipients are closely monitored and followed up; however, upon investigating the reasons behind the patient's lapse in follow‐up care, it was determined that cross‐border tensions between Pakistan and Afghanistan had contributed to his inability to attend scheduled appointments.

## 3 Discussion

DJ stents have revolutionized the field of urology due to their proven efficacy in maintaining renal drainage after surgical procedures. However, the widespread use of these stents has also led to an increased burden of stent‐related morbidity in urology wards around the world [6]. If left unnoticed, various complications can arise, including stent breakage, stone formation, encrustation, migration of stent components, and obstruction. The concern is particularly heightened when a stent is forgotten in a solitary functioning kidney, such as in kidney transplant recipients. These patients often present with symptoms like fever, hematuria, pyuria, and urinary retention. Initial investigations typically include ultrasound and X‐rays to evaluate the presence of a trapped or encrusted DJ stent. In some cases, a computed tomography (CT) scan may be required for a comprehensive assessment before attempting to remove the stent [7].

There have been several reported cases of forgotten DJ stents in renal allograft recipients. For instance, Alwesali et al. [8] described a case involving a patient who had a forgotten DJ stent for 19 years. The patient presented with colicky abdominal pain and recurrent urinary tract infections. The stent was successfully removed using various modalities, including extracorporeal shock wave lithotripsy and transureteral laser lithotripsy. Similarly, Azis et al. [9] detailed an intriguing case of a forgotten DJ stent in an allograft kidney that remained in place for approximately one year. This patient presented with abdominal pain and dysuria. Following a comprehensive radiological evaluation, the DJ stent was removed via ultrasound‐guided mini‐percutaneous nephrolithotomy (mini‐PCNL) utilizing a 19 French rigid nephroscope.

Bardapure et al. [10] reported three cases of renal allograft DJ stent entrapment, with the stents remaining in situ for approximately 3–5 years. All patients presented with lower urinary tract symptoms and recurrent urinary tract infections. In these cases, the stents were removed through cystoscopy; however, one case required two sessions of extracorporeal shock wave lithotripsy for successful retrieval. In another report, Somani et al. [11] documented a case involving a renal allograft with an overlooked DJ stent that had remained in place for about 6 months. The patient presented with worsening renal function. Abdominal ultrasonography revealed a heavily encrusted DJ stent, which was completely removed using a combination of percutaneous nephrolithotomy and cystolitholapaxy. Additionally, Lasaponara et al. [12] described a case where a DJ stent was forgotten for approximately 8 years in an allograft. The stent proved difficult to remove through minimally invasive techniques, ultimately necessitating an open surgical procedure accompanied by uretero–ureteral anastomosis.

Over time, numerous cases of forgotten DJ stents have been reported in the literature, which significantly increases the workload of renal transplant departments within healthcare facilities. Not all forgotten stents can be removed easily; some cases necessitate a multimodal approach and the application of advanced urological technologies for successful retrieval (Table 1).

**TABLE 1:** Comparison of previous studies.

Journal	Year	Author	Presentation	Duration of retention	Modality used
Urology case reports [8]	2022	Alwesali et al.	Colicky abdominal pain and diarrhea	13 years	Nephrostomy
Pan African medical journal [9]	2023	Azis et al.	Abdominal pain and dysuria	1 year	USG guided mini PCNL with 19 Fr rigid nephroscope
Saudi journal of kidney disease and transplantation [10]	2014	Bardapure et al.	2 cases with UTI and one with hematuria	2 cases for 3 years and one for 5 years	2 cases dealt with cystoscopy and one needed two sessions of ESWL
Clinical images in urology [11]	2008	Somani et al.	Deranged RFTS	6 months	PCNL+cystolitholapaxy
Urologia [12]	2013	Lasaponara et al.	UTI	8 years	Uretero‐ureteral anastmosis
Transplantation Proceedings [13]	2004	Yenicesu et al.	Hematuria and dysuria	7 years	Cystoscopy
Cases journal [14]	2009	Bhuva et al.	Nocturia, poor urinary stream	10 years	Ureteroscopy
Annals of RCS England [15]	2024	Gosein et al.	Hematuria	19 years	Flexible cystoscopy +dj stent removal under local anesthesia
Urologia international [16]	2014	Wu et al.	Recurrent UTI	19 years	ESWL+ trans‐ureteral lithotripsy
Transplant international [17]	2005	Romanowsky et al.	Recurrent UTI	4 years	PCNL+Ultrasonic lithotripsy
Kidney international [18]	2007	Lam et al.	UTI	15 years	PCNL +Trans‐ureteral lithotripsy
Clinical Transplantation [19]	2009	Veltman et al.	UTI	5 months	PCNL+ cystolitholapaxy
Journal of CPSP [20]	2012	Lai et al.	Hematuria	5 years	Rigid ureteroscopy

## 4 Conclusion and Results

Cases of forgotten or neglected in situ DJ stents have been reported multiple times worldwide. In developing countries, individuals from rural and lower socioeconomic backgrounds often struggle to comply with their physicians' recommendations. This noncompliance is frequently due to extreme poverty, which can prevent them from traveling to urban centers for follow‐up care. Additionally, factors such as inadequate counseling from healthcare providers and communication barriers stemming from diverse ethnic backgrounds and language differences further complicate the situation. The occurrence of forgotten DJ stents can be mitigated by ensuring effective adherence to surgeons' instructions and fostering a stronger understanding between patients and physicians. By improving communication and education, we can enhance healthcare quality and compliance, ultimately reducing the incidence of such cases globally.

## Author Contributions

**Rao Nouman Ali:** conceptualization, project administration, supervision, visualization, writing – original draft, writing – review and editing. **Adeel Anwaar:** conceptualization, project administration, supervision, visualization, writing – original draft, writing – review and editing. **Wajiha Irfan:** project administration, supervision, visualization, writing – original draft, writing – review and editing. **Farooq Hameed:** project administration, supervision, validation, visualization, writing – original draft, writing – review and editing. **Inam Ul Haq:** data curation, visualization, writing – original draft, writing – review and editing. **Riyan Imtiaz Karamat:** validation, visualization, writing – original draft, writing – review and editing. **Aymar Akilimali:** validation, visualization, writing – original draft, writing – review and editing.

## Ethics Statement

This is a case report utilizing anonymized patient information and so was classified as exempt from review by the Institutional Review Board.

## Consent

A written informed consent was obtained from the patient based on the journal's policies.

## Conflicts of Interest

The authors declare no conflicts of interest.

## Data Availability

The data that support the findings of this study are available on request from the corresponding author. The data are not publicly available due to privacy or ethical restrictions.

## References

[1] M. Al‐Hajjaj , O. A. Alam , B. Abu‐Hussein , and H. A. Muhammad Al Husein , “Forgotten Double‐J Ureteral Stent: An Analysis of 25 Cases in a Tertiary Hospital,” Annals of Medicine and Surgery 80 (2012): 104223, 10.1016/j.amsu.2022.104223. PMC9422216 36045835

[2] S. Patil , K. Raghuvanshi , D. K. Jain , and A. Raval , “Forgotten Ureteral Double‐J Stents and Related Complications: A Real‐World Experience,” African Journal of Urology 26 (2020): 8, 10.1186/s12301-020-0020-3.

[3] R. N. Ali , S. Irfan , W. Irfan , A. Anwaar , M. Irfan , and A. Munim Khan , “Navigating the Triple Threat: Management of Post‐ESWL Urinoma, Steinstrasse, and Obstructive Uropathy: A Challenging Case Report,” Radiology Case Reports 19, no. 12 (2024): 6642–6647. 39403080 10.1016/j.radcr.2024.09.063 PMC11472421

[4] F. Friedersdorff , S. Weinberger , N. Biernath , H. Plage , H. Cash , and N. el‐Bandar , “The Ureter in the Kidney Transplant Setting: Ureteroneocystostomy Surgical Options, Double‐J Stent Considerations and Management of Related Complications,” Current Urology Reports 21 (2020): 3, 10.1007/s11934-020-0956-7. 31960193

[5] S. Alam , N. Ramasamy , C. Thirunavukkarasu , and N. Kumaresan , “Tubeless Percutaneous Nephrolithotomy (PCNL) for Forgotten and Retained Stent in Renal Allograft Recipient: An Interesting Case Report and Lessons Learnt,” BMJ Case Reports 14, no. 1 (2021): e238438, 10.1136/bcr-2020-238438. PMC7849896 33514620

[6] I. D. Kumsa , A. L. Gebreamlak , M. M. Leul , N. B. Hussen , and M. C. Enawgaw , “A Case Report on the Management of Neglected and Forgotten DJ Stent for 15 Years With Severe Encrustation and Multiple Renal and Bladder Stones,” International Journal of Surgery Case Reports 103 (2023): 107859, 10.1016/j.ijscr.2022.107859. 36630763 PMC9841020

[7] S. Agarwal , R. Sarpal , P. Pathak , et al., “Tricks and Tacks in the Management of the Forgotten Double J Stent,” International Surgery Journal 5, no. 3 (2018): 792, 10.18203/2349-2902.isj20180447.

[8] S. M. Alwesali , “A Long Forgotten Ureteral Stent for 13 Years Post Renal Transplantation,” Urology Case Reports 44 (2022): 102156, 10.1016/j.eucr.2022.102156. 35832860 PMC9272339

[9] A. Azis , S. Bakri , M. Z. D. A. Putra , and I. Soehardjo , “Percutaneous Nephrolithotomy for Management Neglected Encrusted Ureteral Stent in a Transplanted Kidney: A Case Report,” Pan African Medical Journal 44, no. 1 (2023): 3, 10.11604/pamj.2023.44.1.37682. 36818034 PMC9935645

[10] M. Bardapure , A. Sharma , and A. Hammad , “Forgotten Ureteric Stents in Renal Transplant Recipients: Three Case Reports,” Saudi Journal of Kidney Diseases and Transplantation: An Official Publication of the Saudi Center for Organ Transplantation, Saudi Arabia 25, no. 1 (2014): 109–112, 10.4103/1319-2442.124514. 24434392

[11] B. K. Somani , A. Todd , and S. Bramwell , “Successful Management of an “Overlooked” Ureteral Stent in a Transplant Kidney,” Urology 72, no. 5 (2008): 1012, 10.1016/j.urology.2008.05.056. 18674805

[12] F. Lasaponara , E. Dalmasso , S. Santià , et al., “Stent Ureterale Dimenticato in Sede Per 8 Anni Dal Trapianto Renale: Trattamento e Follow‐Up a Distanza [A 8‐Year‐Forgotten Ureteral Stent After Kidney Transplantation: Treatment and Long‐Term Follow‐Up],” Urologia 80, no. 1 (2013): 80–82, 10.5301/RU.2013.10743. 23423682

[13] M. Yenicesu , E. Aydur , I. Yildirim , F. Yenicesu , and B. Seckin , “A Long‐Forgotten Indwelling Ureteral Stent in a Renal Transplant Patient,” Transplantation Proceedings 36, no. 5 (2004): 1395–1397, 10.1016/j.transproceed.2004.05.077. 15251341

[14] S. Bhuva , S. J. Kennish , and T. M. Wah , “Forgotten Indwelling Stent in a Transplanted Kidney: A Case Report,” Cases Journal 2 (2009): 27, 10.1186/1757-1626-2-27. 19133113 PMC2639559

[15] S. S. Gosein , J. A. Forster , and J. F. Bolton , “Nineteen‐Year Forgotten Ureteral Stent Removed Under Local Anaesthetic From a Transplanted Kidney,” Annals of the Royal College of Surgeons of England 107 (2025): 528–530, 10.1308/rcsann.2024.0066. 39315954 PMC12400456

[16] F. M. W. Wu , M. Lim , Z. Deng , C. T. Heng , and H. Y. Tiong , “Successful Endourological Management of the ‘forgotten’ Stent in a Transplanted Kidney,” Urologia Internationalis 92, no. 3 (2014): 373–376, 10.1159/000354936. 24458029

[17] I. Romanowsky , L. Lupu , L. Lismer , L. Babaev , E. Z. Neulander , and J. Kaneti , “Percutaneous Nephrolithotomy in Transplanted Kidney—Forgotten Stent With Complete Staghorn and Large Bladder Stone. Case Report,” Transplant International 17 (2005): 877–879, 10.1007/s00147-004-0800-x. 15703922

[18] M. Lam , “Long‐Neglected Stent in a Transplanted Kidney,” Kidney International 71, no. 1 (2007): 5, 10.1038/sj.ki.5001814. 17167502

[19] Y. Veltman , J. M. Shields , G. Ciancio , and V. G. Bird , “Percutaneous Nephrolithotomy and Cystolithalapaxy for a “Forgotten” Stent in a Transplant Kidney: Case Report and Literature Review,” Clinical Transplantation 24, no. 1 (2010): 112–117, 10.1111/j.1399-0012.2009.01133.x. 19925476

[20] D. Lai , Y. He , Y. Dai , T. Li , M. Chen , and X. Li , “A Long‐Forgotten Indwelling Single‐J Stent in a Transplant Kidney,” Journal of the College of Physicians and Surgeons–Pakistan: JCPSP 24, no. Suppl 2 (2014): S152–S154. 24906274

